# Extrapulmonary transport of MWCNT following inhalation exposure

**DOI:** 10.1186/1743-8977-10-38

**Published:** 2013-08-09

**Authors:** Robert R Mercer, James F Scabilloni, Ann F Hubbs, Liying Wang, Lori A Battelli, Walter McKinney, Vincent Castranova, Dale W Porter

**Affiliations:** 1 Pathology and Physiology Research Branch, HELD, NIOSH, Morgantown, WV, USA; 2 Department of Physiology and Pharmacology, West Virginia University, Morgantown, WV, USA

## Abstract

**Background:**

Inhalation exposure studies of mice were conducted to determine if multi-walled carbon nanotubes (MWCNT) distribute to the tracheobronchial lymphatics, parietal pleura, respiratory musculature and/or extrapulmonary organs. Male C57BL/6 J mice were exposed in a whole-body inhalation system to a 5 mg/m^3^ MWCNT aerosol for 5 hours/day for 12 days (4 times/week for 3 weeks, lung burden 28.1 ug/lung). At 1 day and 336 days after the 12 day exposure period, mice were anesthetized and lungs, lymph nodes and extrapulmonary tissues were preserved by whole body vascular perfusion of paraformaldehyde while the lungs were inflated with air. Separate, clean-air control groups were studied at 1 day and 336 days post-exposure. Sirius Red stained sections from lung, tracheobronchial lymph nodes, diaphragm, chest wall, heart, brain, kidney and liver were analyzed. Enhanced darkfield microscopy and morphometric methods were used to detect and count MWCNT in tissue sections. Counts in tissue sections were expressed as number of MWCNT per g of tissue and as a percentage of total lung burden (Mean ± S.E., N = 8 mice per group). MWCNT burden in tracheobronchial lymph nodes was determined separately based on the volume density in the lymph nodes relative to the volume density in the lungs. Field emission scanning electron microscopy (FESEM) was used to examine MWCNT structure in the various tissues.

**Results:**

Tracheobronchial lymph nodes were found to contain 1.08 and 7.34 percent of the lung burden at 1 day and 336 days post-exposure, respectively. Although agglomerates account for approximately 54% of lung burden, only singlet MWCNT were observed in the diaphragm, chest wall, liver, kidney, heart and brain. At one day post exposure, the average length of singlet MWCNT in liver and kidney, was comparable to that of singlet MWCNT in the lungs 8.2 ± 0.3 versus 7.5 ± 0.4 um, respectively. On average, there were 15,371 and 109,885 fibers per gram in liver, kidney, heart and brain at 1 day and 336 days post-exposure, respectively. The burden of singlet MWCNT in the lymph nodes, diaphragm, chest wall and extrapulmonary organs at 336 days post-exposure was significantly higher than at 1 day post-exposure.

**Conclusions:**

Inhaled MWCNT, which deposit in the lungs, are transported to the parietal pleura, the respiratory musculature, liver, kidney, heart and brain in a singlet form and accumulate with time following exposure. The tracheobronchial lymph nodes contain high levels of MWCNT following exposure and further accumulate over nearly a year to levels that are a significant fraction of the lung burden 1 day post-exposure.

## Background

With the wide spread development of commercial carbon nanotube manufacturing and commercial application, carbon nanotubes (CNT) such as MWCNT are an important category of nanoparticle for health risk assessment. There is a need to address the associated bioactivity of these newly manufactured materials. Initial studies focused on the respiratory effects of pulmonary exposure but were limited by the lack of knowledge concerning the occupational levels. Ongoing studies of environments where worker exposure may occur are providing the necessary data to provide realistic inhalation exposures necessary for health risk assessments [1].

MWCNT aspiration exposures in mice conducted at lung burdens relevant to measured occupational exposures have demonstrated early dose- and time-dependent pulmonary inflammation and damage [2]. MWCNT aerosol exposures have demonstrated a variety of effects such as thickening of the alveolar septa [3], severe airway fibrosis in sensitized mice [4], and diffuse histiocytic and neutrophilic inflammation with persistent pulmonary inflammation and granulomas [5].

The small size, lipophilic nature and reported occurrence in the visceral pleura and pleural space [2,6-8] indicate that MWCNT may disseminate elsewhere in the body following pulmonary exposure. Interest in detection of this potential extrapulmonary transport has been increased by reports demonstrating extrapulmonary effects of CNT in the brain [9] and cardiovascular system [10-12] as well as demonstrations that CNT have genotoxic effects [13] and activate several carcinogenic-related signaling pathways [14].

Traditional studies of extrapulmonary transport have been based on detection of a tracer label, typically radioactive [15-17], fluorescent [18,19] or a unique elemental form such as colloidal gold [20] which can be detected by inductively couple plasma mass spectrometry or neutron activation studies. The limits of detection for these methods are typically set either by the instability of the tracer label, instrument sensitivity or the elemental nature of the tracer. For instance, neutron activation analysis has detection limits ranging from a few micrograms per gram of tissue to nanogram levels [21,22]. Detection limits by neutron activation depend on suitable elemental composition and are a function of the element and background interference. While potentially very sensitive, these methods do not generally allow microscopic visualization of individual particles or particle-tissue interactions.

Developments in microscope technology such as enhanced darkfield microscopy allow the direct detection and imaging of singlet CNT. Enhanced darkfield microscopy has been applied for detection and analysis of nanoparticles within the lungs from a variety of exposure [23-25]. We have recently applied enhanced darkfield microscopy techniques to detection of MWCNT in extrapulmonary organs following a 1 day inhalation exposure. That study demonstrated MWCNT translocation to extrapulmonary organs, detecting MWCNT in all the extrapulmonary organs sampled (liver, kidney and heart) within 24 hours of exposure [26] In this report, we extended those observations to a chronic post-exposure study to include a more extensive sampling of sites including the tracheobronchial lymph nodes, the diaphragm, chest wall and brain in addition to the liver, kidney and heart. In addition, we compared the distribution in these sites at 1 day and 336 days after the termination of exposure in order to determine if there was a significant accumulation of MWCNT in systemic tissue with time post-exposure.

## Results

**Figure 1:**
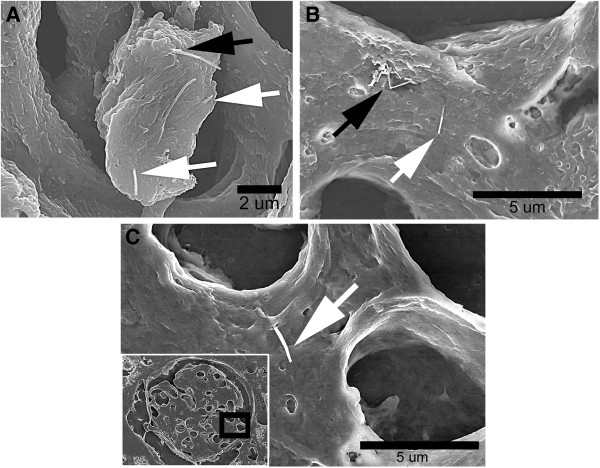
**FESEM examples of MWCNT in lung and kidney tissue sections after MWCNT inhalation exposure.** FESEM image of alveolar macrophages 1 day post-exposure in Figure 1**A** shows typical examples of MWCNT fibers protruding from the surface of alveolar macrophages (black arrow multiple fibers, white arrows singlets). Examples of MWCNT found in the alveolar interstitial space are shown in Figure 1**B** (336 day post-exposure). The image of a kidney section in Figure 1**C** shows a typical example of the single MWCNT found in extrapulmonary organs (336 day post-exposure). The location of this MWCNT singlet within Bowman’s capsule of the kidney is shown by the black rectangle given in the low magnification insert in the lower left of the figure.

FESEM was used to examine MWCNT structures in the lungs and other tissues of this study. Figure 1 shows examples of MWCNT structures in the lungs and kidney. The top two micrographs (Figure 1A and 1B) show MWCNT structures in an alveolar macrophage and the alveolar interstitial space, respectively. As we have previously demonstrated, alveolar macrophages and the interstitial space are the two principal sites for lung burden following inhalation exposure [27]. The surface of the alveolar macrophage in Figure 1A shows numerous protruding MWCNT fibers with white arrows indicating protruding singlets, and multiple MWCNT fibers protruding from the surface indicated by the black arrow at 1 day post-exposure. The black arrow in Figure 1B shows a multiple fiber MWCNT structure just beneath the alveolar epithelium (black arrow) and a singlet MWCNT in the alveolar interstitium (white arrow) at 336 days post-exposure. An example of a singlet MWCNT in the kidney at 336 days post-exposure is shown in Figure 1C with a lower magnification inset indicating the singlet’s position within Bowman’s capsule of the kidney shown by the dark rectangle in the lower left of the micrograph. No systemic pathology was noted in tissue sections used to quantify MWCNT translocation.

**Figure 2:**
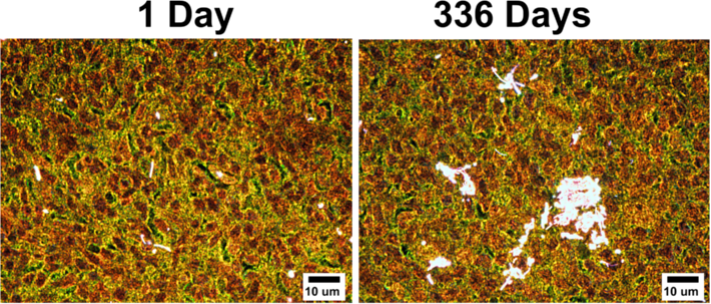
**Enhanced dark-field images of tissue sections from tracheobronchial lymph nodes 1 and 336 days after MWCNT inhalation exposure.** As typified by the micrograph, at 1 day post-exposure singlet MWCNT fibers were observed scattered throughout sections of lymph nodes. However, 336 days post-exposure, numerous dense concentrations of MWCNT fibers were found within the lymph nodes. MWCNT fibers are bright white in these enhanced darkfield images due to scattering of light by MWCNT, while cell nuclei are brownish red and other tissue elements are green.

As illustrated by the representative, enhanced darkfield images of tracheobronchial lymph nodes in Figure 2, there were significant accumulation of and changes in the distribution of MWCNT fibers within the lymph nodes between 1 and 336 days exposure. At 1 day post-exposure MWCNT fibers in the tracheobronchial lymph nodes were generally singlet MWCNT or small MWCNT agglomerates which were widely scattered throughout the cortex. With increasing days after exposure, foci of phagocytic cells were associated with dense clusters of nanotubes within the tracheobronchial lymph nodes. Translocation of MWCNT from the lung to the tracheobronchial lymph node has previously been demonstrated in rats [28]. In the mouse, we have previously described MWCNT translocation to the tracheobronchial lymph nodes after acute MWCNT inhalation [27]. After acute exposure, the MWCNT in the lymph node were predominantly localized to the deep paracortical region within cells morphologically consistent with macrophages and dendritic cells. Cells containing both MWCNT and apoptotic debris are also observed. In addition to similar changes noted in our previous acute inhalation study, the tracheobronchial lymph nodes at 336 days were enlarged by 70% above their clean-air counterparts, dark gray in their appearance due to the accumulation of MWCNT and contained the foci of dense MWCNT aggregates noted above.

**Figure 3:**
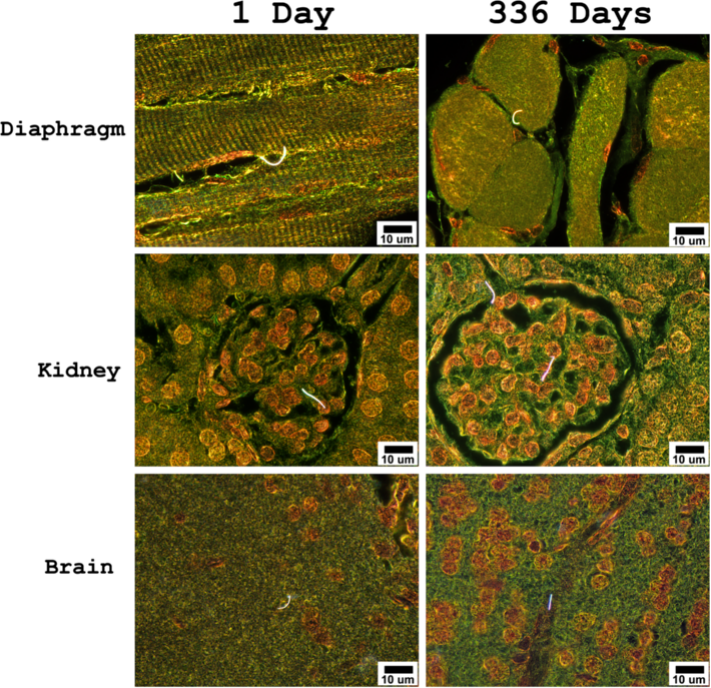
**Enhanced darkfield images of MWCNT fibers in the diaphragm, kidney and brain at 1 day and 336 days after inhalation exposure.** MWCNT fibers in these figures are bright white, cell nuclei are brownish red and other tissue elements are green. With rare exceptions, MWCNT fibers detected in extrapulmonary organs were singlets. Normal (transmitted) light was blended into the fields and contrast adjusted to make the tissue histology of the organs visible in these photographs.

Examples of the enhanced darkfield images of MWCNT fibers in the diaphragm, kidney and brain at 1 day and 336 days after inhalation exposure are given in Figure 3. MWCNT fibers detected in the diaphragm, chest wall and extrapulmonary organs were with rare exceptions, singlets. In these tissues, approximately one in 200 MWCNT structures were doublets. The nearly exclusive observation of singlet MWCNT in diaphragm, chest wall and extrapulmonary organs was in contrast to the observations of dense, large MWCNT structures observed at 336 days post-exposure in the tracheobronchial lymph nodes (Figure 2). At 336 days post-exposure, the concentration of fibers in diaphragm and extrapulmonary organs (Table 1) was significantly higher than at one day post-exposure. At 336 days post-exposure, singlet MWCNT were detectable in nearly all 40x fields of view in sections of liver and kidney with multiple, but separated, fibers detected in the same field of view as shown in the figure for the kidney. Average fiber length was 8.4 ± 0.3 and 8.8 ± 0.3 um at 1 and 336 days post-exposure in extrapulmonary organs (Mean ± S.E., N = 100). In the lungs, average fiber length of singlets was 8.2 ± 0.3 and 7.5 ± 0.4 um at 1 and 336 days post-exposure (N = 100).

**Table 1:** Extrapulmonary MWCNT concentration, number

**Day post-inhalation**^**1**^	**Organ**	**Weight gm**	**Concentration #/gm tissue**	**Total #**	**% of lung burden**^**2**^
**1**	Lymph Nodes	0.03	508,776,984	14,263,560	1.08000
	Liver	1.53	25,767	39,303	0.00281
	Kidney	0.45	16,639	7,438	0.00105
	Heart	0.12	11.849	1,371	0.00022
	Brain	0.38	7,231	2,776	0.00101
	Chest Wall	1.00	13,774	13,764	0.00354
	Diaphragm	0.07	6,767	507	0.00025
				Total%	1.089%
**336**	Lymph Nodes	0.05	1,807,273,684	84,845,500	7.30000
	Liver	1.83	196,798	360,456	0.02740
	Kidney	0.54	126,973	68,165	0.00520
	Heart	0.14	62,401	8,670	0.00070
	Brain	0.46	53,569	24,740	0.00190
	Chest Wall	1.07	10,382	12,457	0.00090
	Diaphragm	0.09	59,915	5,392	0.00040
				Total%	7.337%

^1^Body weights of MWCNT exposed mice were 22.9 ± 0.5 and 40.5 ± 2.2 g at 1 day and 336 days post-exposure, respectively.

^2^100 × Total number of fibers in organ divided by 1 day post-exposure lung burden of 1,321 million fibers per lung.

**Figure 4:**
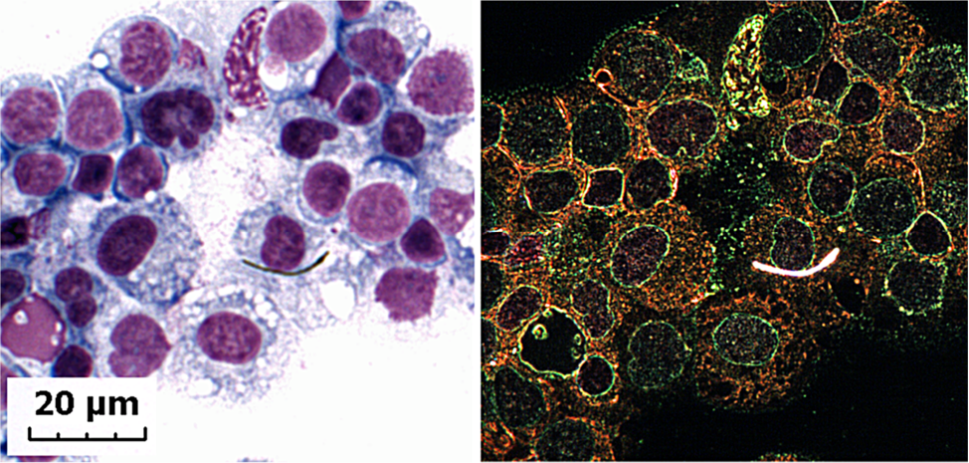
**Light and enhanced darkfield micrographs of MWCNTs detected in lavage of pleural space.** The figure shows a comparison of the light and enhanced darkfield image of a singlet MWCNT in lavage of the pleural space in mice at 336 days post-exposure.

Analysis demonstrated that singlet MWCNT were present in lavage of the pleural space from MWCNT-exposed mice at 336 days post-exposure. Figure 4 shows an example of same singlet MWCNT in the lavage fluid as visualized by light and enhanced darkfield microscopy. MWCNTs in the pleural lavage were singlets with an average length of 6.9 microns. Singlet MWCNTs in the lavage of the pleural space were found either in close contact or penetrating into the cytoplasm and/or nucleus of monocytes in the pleural lavage. There was approximately 1 singlet MWCNT per 3000 cells in the pleural lavage. This is comparable to the 1 crocidolite fiber per 4000 cells in pleural lavage reported for an inhalation exposure study of asbestos-induced pleural injury in rats [29]. Based on counting cytocentrifuge slides of the 1 ml pleural lavage fluid there was an average of 23.7 ± 7.6 (Mean ± S.E., N = 5) singlet MWCNT, in the lavage of the pleural space. Pleural lavage at 1 day post-exposure was not conducted.

The light micrograph of the same singlet MWCNT on the left side of Figure 4 was only visible over a narrow range of focus and was very difficult to identify even though nearby cells, visible in enhanced darkfield imaging, were clearly visible in the light microscope. The exercise of identifying singlet MWCNT in lavage fluid from the pleural space, and the example of side-by-side comparison of enhanced darkfield versus light microscopy of MWCNT structures containing a few or singlet MWCNT structures indicate that enhanced darkfield is the optimal instrument for scanning of wide fields to detect nanoparticles. Based on side-by-side comparisons, the transmitted light microscope is not a reliable tool for identification of these MWCNT structures.

**Figure 5:**
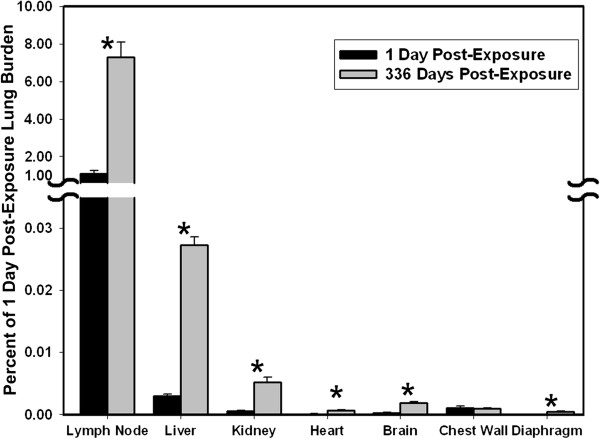
**Percentage of 1 day post-exposure lung burden detected in tracheobronchial lymph nodes, extrapulmonary organs, diaphragm and chest wall.** By 336 days post-exposure there was a 7-fold increase in MWCNT in extrapulmonary organs and diaphragm over that measured at 1 day post-exposure. Categories of extrapulmonary organs are ordered relative to MWCNT concentration in the respective tissue. Asterisks indicate significantly different between day 1 and 336 days post-exposure, p < 0.05.

Results from morphometric analysis of MWCNT fibers in tracheobronchial lymph nodes, diaphragm, chest wall and extrapulmonary organs are shown in Figure 5. At 1 day post-exposure, the content of MWCNT fibers in the tracheobronchial lymph nodes was principally in the form of singlets or a few fibers per MWCNT structure and accounted for 1.08% of the lung burden 1 day post-exposure. MWCNT structures in tracheobronchial lymph nodes at 336 days included foci with dense accumulations with the content being substantially increased and equal to 7.34% of the lung burden 1 day post-exposure. MWCNT were only rarely observed in examination of the adventitia attached to the node capsules.

The high level of MWCNT burden delivered to the tracheobronchial lymph nodes was approximately 300 times greater than the burden delivered to the liver which was the highest observed in extra-pulmonary organs. Total MWCNT burden for diaphragm, chest wall and extrapulmonary organs was 0.009 and 0.037 percent of lung burden at 1 day and 336 days, respectively, after exposure. By 336 days post-exposure there was a 6 to 7-fold increase in the concentration of MWCNT in extrapulmonary organs and diaphragm, excluding the chest wall. MWCNT in the chest wall did not change significantly over this period.

The chest wall, which had a concentration of fibers comparable to that of the overall median concentration for extrapulmonary organs at 1 day post exposure did not accumulate fibers with time post-exposure as was observed for other organs. At the same time, the diaphragm, which is the other major pleural opposed surface, demonstrated an approximate 7-fold increase similar to the other organs examined. The chest wall, unlike other tissues, was decalcified prior to embedding and sectioning. This difference in treatment could be responsible. To test for a treatment effect, weighed samples of the MWCNT were treated extensively with the decalcification solution in the same manner as the chest wall tissue and then washed, dried and reweighed. In FESEM examination of the formic acid-treated MWCNT fibers, no apparent degradation due to the treatment was observed. Average length of MWCNT fibrils in the chest wall section was not significantly different than that of the diaphragm which was not decalcified with formic acid.

In Table 1 are given the organ weights, concentration of MWCNT fibers per gram of tissue, total MWCNT fibers in each tissue and the percentage relative to the lung burden at 1 day post-exposure for the tracheobronchial lymph nodes, diaphragm, chest wall and extrapulmonary organs. Excluding the lymph nodes and respiratory muscles, the concentrations at 1 day post-exposure varied by a factor of four with the highest concentration in the liver at 25,767 fibers/g and the lowest in the brain at 7,231 fibers/g. At 1 day post-exposure the order in terms of fibers per gram was liver, kidney, heart, and brain with the liver concentration being significantly elevated above the other organs. This does not appear to follow the order that is found in the distribution of blood flow per ml min^-1^ g^-1^ which has been reported to be heart, kidney, brain and liver based on radiolabeled microspheres in anesthetized mice [30]. Enhanced uptake of MWCNT by the reticuloendothelial system of the liver, fenestrated endothelium of the kidney and restricted uptake due to the blood–brain-barrier in the brain may account for these differences between organ blood flow and MWCNT uptake.

## Discussion

Recent developments in microscope technology, such as enhanced darkfield microscopy, allow the direct detection and imaging of singlet CNT. The ability to detect a single fiber in a large tissue section affords a very high detection limit. For instance, a typical animal inhalation exposure at 5 mg/m^3^ contains 1.4 × 10^10^ particles [31]. On average, a singlet MWCNT fiber of this aerosol would weigh 0.4 picograms. A typical section from a mouse is approximately 2 cm by 2 cm by 0.0005 cm deep or 0.002 cm^3^. Assuming the detection limit is one fiber per section, identification of a singlet MWCNT in a tissue section represents the ability to detect less than a nanogram of fibers per gram of tissue. Enhanced darkfield microscopy has been applied for detection and analysis of nanoparticles within the lungs from a variety of exposure [23-25]. In the present study, based on Table 1, the percentage of lung burden transported to the diaphragm at 1 day post-exposure was 0.00025 percent. This is equivalent to detection of 3 singlet MWCNT in the diaphragm for every million MWCNT fibers initially deposited in the lungs.

We have previously shown that exposure to MWCNT results in significant fiber accumulations within the interstitial spaces of the lungs, the lymphatics and the visceral pleura [2,6,27,32]. The MWCNT structures within these sites were generally composed of single fibers or structures containing a few fibers, while MWCNT structures with a greater number of fibers remained in the airspaces and within alveolar macrophages of the lungs. The present study extends those results to demonstrate that MWCNT deposited in the lungs are transported to the pleura and/or extrapulmonary organs. The magnitude of extrapulmonary transport can be evaluated by expressing the lung burden transported in terms of fiber number translocated to the extrapulmonary organs (liver, kidney, hear and brain) relative to the initial number of fibers deposited in the lungs at 1 day post-exposure. Expressing the results of Table 1 in that manner, approximately 1 fiber deposits in an extrapulmonary organ for every 25,700 MWCNT fibers of lung burden present in the lungs at 1 day post-exposure after a 12 day exposure period. Transport during the post-exposure period from 1 day to 336 days significantly elevated the accumulation in extrapulmonary tissues with approximately 1 fiber for every 2,800 fibers present in the lungs at 1 day post-exposure being transported to extra-pulmonary tissues.

The significance of these levels of transport depends on the critical target for health risk assessment. At the cellular level, the mouse lung [33] and liver [34] contain approximately 119 billion and 15 billion cells, respectively. Dividing these cell numbers by the respective fiber counts (Table 1) demonstrates that, in the lungs, at 1 day post-exposure there is approximately 1 MWCNT fiber per 90 lung cells. In the liver at 1 day post-exposure, there is approximately 1 fiber per 381,650 liver cells and 1 fiber per 41,600 liver cells at 336 days post-exposure. These results clearly demonstrate that mass-action effects such as fibrosis are unlikely in extrapulmonary organs at these levels of transport. On the other hand, recent studies [13] have demonstrated that MWCNT fibers have highly efficient mutagenic effects which could prove to be a health risk concern for neoplastic development.

Donaldson et al. [35] raised concern that high aspect ratio CNT may, like asbestos, induce mesothelioma. Indeed, Takagi et al. [36] reported that intraperitoneal injection of MWCNT (3 ug/mouse) led to mesothelioma of the abdominal wall. Murphey et al. [37] reported persistent inflammation and fibrosis of the parietal pleural surface 24 weeks after intrapleural injection of long (>15 um) but not short (<4 um) MWCNT (5 ug/mouse). Therefore, there is great interest in determining whether pulmonary exposure to MWCNT leads to migration of MWCNT into the pleura, quantifying the magnitude of this migration, and determing if pleural lesions result. Ryman-Rasmussen et al. [8] reported MWCNT in subpleural lung tissue after inhalation exposure in mice. Porter et al. [2] were the first to demonstrate that such subpleural MWCNT can pierce the surface of the lungs and enter the intrapleural space after aspiration of these nanoparticles. Mercer et al. [6] conducted quantitative microscopic morphometry of lung tissue from the Porter et al. study and reported 12,000 penetrations of MWCNT into the intrapleural space 2 months after aspiration of MWCNT (80 ug/mouse). The current study extends these results in the following manner: 1) lungs were exposed to MWCNT by inhalation of mice over 12 days to yield a lung burden of 28 ug/lung, 2) morphometric analysis of chest wall and diaphramattic tissue was conducted at 1 and 336 days post-exposure, and 3) the number of MWCNT in the ches wall and diaphragm was quantified. Results indicate that at 336 days after inhalation exposure 12,457 fibers were found in the chest wall while 5,292 fibers were found in the diaphragm. Whether this fiber burden in pleural tissue results in inflammation, lesions and/or transformation of mesothelial cells is currently being investigated in an ongoing inhalation study at NIOSH.

Recently, Schinwald et al. [38] determined the threshold fiber length which would result in pleural inflammation 1 week after intrapleural injection of silver nanofibers of well defined lengths. Results showed a distinct length threshold, with fibers longer than 4 um leading to a pathogenic response. The MWCNT fibers aerosolized in the current study have a mean length of 4.3 um [31]. Of interest, singlet MWCNT found in the chest wall, diaphragm, and systemic tissue appear to be in the 7–8 um range. Although these fibers should be too short to cause frustrated phagocytosis, they appear to be long enough to be retained in the pleura as demonstrated for MWCNT harvested by pleural lavage 336 days post-exposure (Figure 4). Therefore, if the pleural burden is sufficiently high a pathogenic response may be expected.

The route by which MWCNT are transported to the extra-pulmonary cannot be directly determined from the results of this study. Given the low levels found in the extrapulmonary organs a number of routes are possible. Phagocytosis of MWCNT by circulating monocytes/macrophages and neutrophils which transiently passage via the circulation, may provide a significant route. Observation of MWCNT loaded circulating cells in the capillary bed of extrapulmonary organs would be expected if phagocytosis or other adherence to MWCNT in the lungs resulted in transport to extrapulmonary organs. The nearly exclusive transport of singlet MWCNT to extrapulmonary organs (Figures 1 and 3) suggests that this route is not significant. Specific attempts were made to identify MWCNT in cells within the hepatic sinuosoids of the liver but none were detected. However, cases which demonstrated singlet MWCNT apparently at, or within, the endothelial boundary of capillaries were observed. An example is shown by the singlet MWCNT in the nearly tangential section through a capillary of the brain in the lower right panel of Figure 3.

MWCNT cleared from the lungs via the macrophage-mucociliary escalator may be reabsorbed by the gastrointestinal tract. In whole-body inhalation exposures, which was the exposure method in our study, intestinal absorption of MWCNT ingested during preening of fur may also be a significant pathway. Reports on the contribution by gut absorption to systemic delivery of nanoparticles have come to differing results. Kreyling et al. [16] found less than 1 percent of deposited nanoparticles transported to systemic organs and did not find evidence for gut absorption of 15 or 80 nm radio-labeled iridium particle when comparing extrapulmonary transport by whole body inhalation, gavage and intratracheal instillation. In a subsequent study by this group, using radiolabelled ultrafine carbon particles, Oberdörster et al. [17] found significant transport to the liver in whole body exposures of the rat and concluded that differences in translocation between the different nanoparticles may reflect differences in chemistry of the particles or gut absorption.

Finally the tracheobronchial lymphatic are a major route for fluid exchange of the lungs. Macrophage mediated transport through the lymphatic network has been shown to be important in particulate clearance from the lungs [39]. The high lymphatic burdens observed 1 day post-exposure and at 336 days post-exposure (Table 1, Figures 2 and 4) indicate that the transport of MWCNT thru the lymphatics and ultimately into the venous circulation may be a major route for systemic delivery of MWCNT. Consistent with this role, dilation of peribronchiolar lymphatics was noted in the present study, as well as our previous acute inhalation exposures [27]. However, direct measurements of MWCNT in the venous outflow from the lymphatics will be necessary to demonstrate the potential significance of this route.

## Conclusions

One day after a 12 day inhalation exposure period MWCNT fibers were found throughout a wide range of lung associated tissues (lymph nodes, chest wall, diaphragm) and in extrapulmonary organs. Over the post-exposure period of 336 days, the lymph nodes accumulated a substantial fraction of 1 day post-exposure lung burden, while the levels in extrapulmonary organs increased approximately 6 to 7 fold. Inhaled MWCNT are capable of wide-spread dissemination and accumulation throughout the body. Outside the lungs and tracheobronchial lymph nodes the levels of accumulation are not likely to pose a risk of fibrosis. However, by 336 days post-exposure, the concentration of MWCNT per gram of tracheobronchial lymph nodes exceeds that in the lungs and is likely to produce adverse reactions, as those reported previously after exposure of mice by aspiration [2].

The slow rate at which MWCNT are cleared from the lungs by normal macrophage-mediated processes coupled with the high concentration in the tracheobronchial lymph nodes and the chronic accumulation in extrapulmonary organs and pleural associated tissue clearly demonstrate the need to address concerns about potential extra-pulmonary health effects from inhalation exposures to MWCNT.

## Methods

### Animal

Male C57BL/6 J mice (7 weeks old) were obtained from Jackson Laboratories (Bar Harbor, ME). Mice were housed one per cage in polycarbonate ventilated cages, which were provided HEPA-filtered air, with fluorescent lighting from 0700 to 1900 hours. Autoclaved Alpha-Dri virgin cellulose chips and hardwood Beta-chips were used as bedding. Mice were monitored to be free of endogenous viral pathogens, parasites, mycoplasms, Helicobacter and CAR Bacillus. Mice were maintained on Harlan Teklad Rodent Diet 7913 (Indianapolis, IN), and tap water was provided ad libitum. Animals were allowed to acclimate for at least 5 days before use. All animals used in this study were housed in an AAALAC-accredited; specific pathogen-free, environmentally controlled facility. All animal procedures were approved by the NIOSH ACUC.

### Carbon nanotube source

MWCNT used in this study were obtained from Hodogaya Chemical Company (MWNT-7, lot #061220-31) and were manufactured using a floating reactant catalytic chemical vapor deposition method followed by high thermal treatment in argon at 2500°C furnace. This lot of MWCNT was fully characterized in our prior report in which acute inhalation exposures were conducted with this lot of MWCNT [27]. Briefly, MWCNT trace metal contamination was 1.32%, with iron (1.06%) being the major metal contaminants. The bulk MWCNT were analyzed using several different techniques; 1) high resolution TEM, 2) XPS, and 3) electron spin resonance spectroscopy and are described in detail [27]. Bulk and aerosol MWCNT morphology, were also analyzed by TEM and SEM in that study and found to have a similarity in particle shape and configuration to those collected from the breathing zones of a MWCNT manufacturing workplace [40].

### MWCNT aerosol generation and aerosol characterization

Mice were exposed to a MWCNT aerosol (5 mg/m^3^, 5 hours/day) for 12 days, using an acoustical-based computer controlled system designed and constructed by our laboratory [41]. Details of the exposure system, aerosol control performance and aerosol characterization have previously been published [27]. In brief, the inhalation exposure system combines air flow controllers, aerosol particle monitors, data acquisition devices, and custom software with automated feedback control to achieve constant and repeatable exposure chamber temperature, relative humidity, pressure, aerosol concentration, and particle size distributions. The generator produces airborne particles continuously for long periods of time, e.g. 35 hours of continuous operation, with minimal fluctuations during an exposure period. The uniformity of test atmosphere in the chamber was evaluated to have a total variation of < 5%. In this study, the MWCNT aerosol mass concentration was continuously monitored with a Data RAM (DR-40000 Thermo Electron Co, Franklin, MA), and gravimetric determinations (37 mm cassettes with 0.45 μm pore-size Teflon filters) were used to calibrate and verify the Data RAM readings. The mass mode aerodynamic diameter was 1.3 μm with a count mode aerodynamic diameter of 0.42 μm [27]. When characterized by lognormal statistics, the distribution was shown to have a mass median aerodynamic diameter (MMAD) of 1.5 μm and a geometric standard deviations (GSD) of 1.67 [27].

In the prior acute MWCNT inhalation study, run concurrently with this chronic study, the MWCNT lung burden in the mouse at 1 day post-exposure was determined to be 28.1 ug/lung [27]. Workplace MWCNT-containing airborne dust levels of approximately 400 μg/m3 have been reported in a research laboratory [40] while a later study reported the highest total particle concentration of 320 μg/m3 with a mean of 106 μg/m3 based on results obtained from monitoring total workplace dust levels at seven MWCNT facilities [42]. Assuming a level of one-tenth of the reported workplace range (10–40 ug/m^3^) Porter et al. [27] demonstrated that human worker exposure to MWCNT, performing light work for approximately 8.5 years in a work environment would be expected to produce a similar concentration of MWCNT in terms of micrograms per square meter of alveolar epithelial surface area in the human worker lungs as this inhalation study produced in the mouse lung. Thus the current mouse exposure represents an approximate feasible human occupational exposures.

#### Pleural lavage

Lavage of the pleural space was conducted prior to the instillation of fixative in an additional group of mice at 336 days post-exposure and in a corresponding clean-air group. Mice were deeply euthanized with an i.p. injection of sodium pentobarbital (>100 mg/kg body weight). For pleural lavage, a midline incision of the abdomen was made and a small cut made in the diaphragm. A blunted plastic tube connect to a 1 ml syringe was then used to slowly infuse and withdraw 1 ml of ice cold Ca^2+^ and Mg^2+^-free phosphate buffered saline, pH 7.4, supplemented with 5.5 mM D-glucose (PBS) into the pleural space 5 times. Slides were prepared from 0.2 ml aliquots using a cytocentrifuge (Shandon Elliot Cytocentrifuge, London). The cytospin preparations were stained with modified Wright-Giemsa stain and MWCNT fibers counted by scanning the entire sample at 60x magnification with an enhanced darkfield microscope.

#### Lung fixation and section preparation

At 1 day and 336 days after the 12 day exposure period, mice were euthanized by an overdose of pentobarbital (>100 mg/kg body weight, i.p.) and lungs and extrapulmonary tissues were preserved by whole body vascular perfusion of paraformaldehyde while the lungs were inflated with air. Separate, clean-air control groups were studied.

For whole body perfusion the trachea was cannulated, the lungs inflated with 1 ml of air and a midline incision of the chest was made to expose the heart and lungs. The left ventricle of the heart was punctured with a large bore needle connected to a reservoir 100 cm above the chest wall of the animal. In quick sucession the right atrium was cut, to allow outflow, the reservoir valve was opened to allow perfusion of the whole body with clearing solution (heparinized saline). After the clearing solution was passed (2 to 5 ml), the reservoir was switched to paraformaldehyde and the whole body perfusion fixed (~ 25 to 50 ml).

Following fixation the tracheobronchial lymph nodes, diaphragm, heart, kidney, liver and brain were removed, and sliced into 5–6 mm thick tissue blocks and embedded in paraffin. The left lung was removed, cut into a coronal mid-section and processed independently to avoid potential for contamination. The chest wall was decalcified in formic acid prior to embedding. Sections (5 micron thick) were collected on ultrasonically cleaned, laser cut slides (Schott North America, Inc, Elmsford, N.Y. 10523) to avoid nanoparticle contamination from the ground edges of traditional slides. To enhance the contrast between tissue and MWCNT, sections were stained with Sirius Red. Sirius Red staining consisted of immersion of the slides in 0.1% Picrosirius solution (100 mg of Sirius Red F3BA in 100 ml of saturated aqueous picric acid, pH 2) for 1 hour followed by washing for 1 minute in 0.01 N HCl. Sections were then briefly counterstained in freshly filtered Mayer’s hematoxylin for 2 minutes, dehydrated, and coverslipped. For serial section analysis to determine the mean caliper diameter of MWCNTs in tissue, five serial sections of the lungs (3 um thick) were collected on one slide and stained as described above.

### Field emission scanning electron microscopy

For scanning electron microscopy, sections of the lung were cut at 8 microns, placed on carbon planchets, deparaffinized and sputter coated. After coating, the specimens were examined with a Hitachi Model S-4800 Field Emission Scanning Electron Microscope (FESEM) at 5 to 10 kV and at working distances of 4.5 mm to 6 mm for magnifications of 100,000× to 1000×, respectively. Photographs were taken in slow scanning mode at 1280 × 1024 pixels. Use of thin sections from paraffin embedded tissue was found to be preferable to large, unevenly cut blocks because it provided a uniform thickness of organic material on the carbon planchet. The 8 micron sections were thick enough to convey three-dimensional information but were also less likely to charge or undergo physical shifts when examined at the high magnifications necessary to study nanomaterials.

#### Enhanced-darkfield light microscopy imaging of nanoparticles

Carbon nanotubes in sections from exposed lungs were assessed using an enhanced-darkfield optical system. Using this method of imaging, lung sections can be easily scanned at relatively low magnification to identify CNTs that would not be detected by other means. Nanomaterials, such as carbon nanotubes, have dimensions less than the wavelength of light, have closely packed atoms, and typically have a refractive index significantly different from that of biologic tissues and/or mounting medium. These characteristics produce significantly greater scattering of light by nanoparticles than by the surrounding tissues. The enhanced-darkfield optical system images light scattered in the section and, thus, nanomaterials in the section stand-out from the surrounding tissues with high contrast. Detection of a nanomaterial in a section thus depends on the ability of the particle to scatter light and the number of scattered photons required for detection by the imaging system.

In practice enhanced darkfield has been found to be an essential tool to detect and measure MWCNT regional pulmonary distribution [32] and in detection of MWCNT systemic transport following inhalation in the rat [26]. Detection and quantitiatve assessment of other nanoparticles has been reported. These include the diesel fuel catalyst, cerium oxide [23], titanium dioxide nanospheres [24], titanium dioxide nanospheres and nanobelts [25] and summarized in a review article on pathologic assessment of nanoparticles which describes aspects of enhanced darkfield in nanoparticle detection [43].

The optical system for enhanced darkfield microscopy consisted of high signal-to-noise, darkfield-based illumination optics adapted to an Olympus BX-41 microscope (CytoViva, Auburn, AL 36830). Sections for dark-field examination were specifically cut from paraffin blocks and collected on ultrasonically cleaned, laser cut slides (Schott North America Inc, Elmsford, N.Y. 10523) to avoid nanoparticle contamination from the ground edges of traditional slides. After staining with Sirius Red-Hematoxylin, sections were coverslipped with Permount. After alignment of the substage oil immersion optics with a 10x objective, sections were examined with 60x or 100x oil immersion objectives. Enhanced darkfield images were taken with a 2048 × 2048 pixel digital camera (Dage-MTI Excel digital camera XLMCT, Michigan City, In 46360).

#### Number of MWCNT fibers in diaphragm, chest wall and extrapulmonary organs

Measurement of the distribution of MWCNT to the diaphragm, chest wall and extrapulmonary organs was made at 1 day and 336 days after the 12 day exposure period by counting the number of fibers in sections of diaphragm, chest wall and extrapulmonary organs. These counts of fibers per unit area were converted to number per organ using the mean caliper diameter of the fibers to convert from fibers per unit area to fibers per unit volume using the same morphometric methods as previously reported for counting of granulomas [44], alveoli [45], and cells/nuclei [33,46]. Use of mean caliper diameter to convert from number per unit area to number per unit volume in a shape independent and unbiased fashion was originally described by Hillard [47]. As illustrated in Table 2, the number of fibers was converted to number per unit area by dividing the counts by the area of the respective section. To determine the number of fibers per unit volume, the number per unit area was divided by the mean caliper diameter (4.5 and 4.2 um for 1 and 336 days post-exposure, respectively) plus section thickness (5 um). Results were expressed as number per gram assuming a unit density of 1 g/cm^3^ for tissue. Mean caliper diameter was measured by taking serial sections and measuring the length of the fiber projection in the z-axis.

**Table 2:** Formula for number of singlet MWCNT in organ

N_a_	Number per unit area cm^2^
D	Mean caliper diameter of singlet MWCNT cm
T	Section thickness cm
V	Organ Volume = Organ Weight gm × 1 cm^3^/gm
N_V_	Number per unit volume #/ cm^3^
**N**_**O**_	Number per organ

N_V_ = N_a/(D+T)_.

N_O_ = N_V_ × V.

Sections of clean-air control lungs were scanned as well for fibers but were negative for the presence of MWCNT fibers. Total MWCNT fiber number of 1,321 million was used for the lung based on previously reported measurements of 1 day post-exposure lung burden of MWCNT fibers of 28.1 ug/lung previously determined for this inhalation study [27] and a conversion of 47 million MWCNT fibers per ug [24].

#### Measurement of singlet MWCNT fiber length in lungs, liver and kidney

Optical sectioning through a series of serial sections in lung, liver and kidney was used to measure the length of singlet MWCNT at 1 and 336 days after the termination of inhalation exposure. Serial sections consisted of 4–5 sections, 5 um thick, mounted on a single slide. For each tissue/series (lungs or systemic organs) the second section of each series was scanned for singlet MWCNT using enhanced darkfield illumination with a high numerical aperture, 100x oil immersion objective. When a singlet MWCNT was identified in the second section, it was selected for measurement if it did not continue down into the first section to produce an unbiased (length independent) sampling. Thus singlet MWCNT were selected for measurement if they began in the second section. In the second section of each selected singlet, the lower-most end was focused on, photographed and the depth (Z co-ordinate on focus knob) recorded. A point mid-fiber in the section (or at a sharp bend/inflection point if present) was next focused, photographed and the depth (Z co-ordinate) recorded. Finally, the upper-most end of the singlet was focused on, photographed and the depth (z co-ordinate) recorded. If the singlet continued in the adjacent upper section(s) the process of focus, photography and Z-co-ordinate measurement was repeated to the end of the singlet. ImageJ was then used to measure the X-Y co-ordinates of the singlet/middle points. The length of the singlet was then calculated from the 3-dimensional distance from one end through the middle to the other end (including adjacent sections if the singlet was not completely contained within a section). Approximately 20 singlet MWCNT were measured in each lung, liver and kidney in 5 animals at 1 and 336 days after the inhalation exposure.

#### Measurement of MWCNT content in tracheobronchial lymph nodes

Direct counting of MWCNT in tracheobronchial lymph nodes was not possible due to the dense aggregations beyond 1 day post-exposure. Instead, the volume density of MWCNT relative to the volume density of MWCNT in the lung at 1 day post-exposure was used to determine the lymph node content. For this purpose, 6–12 photographs were taken at 60x uniformly distributed across the mid-section profile of the capsule of each lymph node and across the mid-section of the left lung. Each section was thresholded to produce white for MWCNT and black for air/tissue and the volume proportion determined using ImageJ. The accuracy of this procedure was verified by direct comparison of a sample of photograph to manual point counting which produced volume densities for MWCNT that were within 1% of the threshold image determined values. The mass of MWCNT in the tracheobronchial lymph nodes was then obtained by multiplying the lung burden times the ratio of volume densities, which was then multiplied by the ratio of tracheobronchial lymph node volume to lung volume.

Direct weighing of the tracheobronchial lymph nodes was not suitable to determine the capsule volume of the nodes where MWCNT were found. Unlike extrapulmonary organs, dissected tracheobronchial lymph nodes have a high mass of associated adventitia which cannot be removed without risk of damage to the nodes where the MWCNT are concentrated. To avoid injury to the capsule, which was also needed for pathological assessment, the entire dissected tracheobronchial lymph node was embedded and a mid-block tissue section photographed. The area of the capsule of the tracheobronchial lymph nodes was then used to calculate the organ weight assuming a tissue density of 1 gm/cm^3^.

### Statistical analyses

Data were analyzed using analysis of variance (STATGRAF). Bartlett’s test was used to test for homogeneity of variances between groups. Statistical differences were determined using one-way analysis of variance with significance set at p ≤ 0.05. When significant F values were obtained, individual means at 336 days post-exposure were compared to corresponding 1 day post-exposure for the corresponding organ using Duncan’s multiple range test [48], and P < 0.05 was considered to be significant. Data are given as Means ± S.E.

## Abbreviations

BET: Brunauer-emmett-teller method; BMD: Benchmark dose; CNT: Carbon nanotubes; DM: Dispersion medium; DPPC: 1,2 dipalmitoyl-sn-glycero-3-phosphocholine; FESEM: Field emission scanning electron microscope; GSD: Geometric standard deviations; MMAD: Mass median aerodynamic diameter; MWCNTs: Multi-walled carbon nanotubes; PBS: Phosphate-buffered saline; TEM: Transmission electron microscope.

## Competing interests

The authors declare that they have no competing interests.

## Authors’ contributions

RM conceived of the study, developed the morphometric methods, conducted the FESEM evaluation, analyzed the experimental results and drafted the manuscript. JS performed the morphometric counting and assisted in analysis of results. AH was involved in the planning and writing of the manuscript. LW contributed to the experimental design and assisted in lung preparation. LB provided important information on sampling of the lungs for study and conducted lung preparation for histopathology. WM developed the MWCNT aerosol generation and exposure systems. VC and DP contributed to the experimental design, acquisition of funding and writing of the manuscript. All authors read and approved the final manuscript.
